# Remote Neuropsychological Testing as an Alternative to Traditional Methods—a Convergent Validity Study

**DOI:** 10.1093/arclin/acaf013

**Published:** 2025-02-20

**Authors:** Emma Wärn, Linus Andersson, Nils Berginström

**Affiliations:** Department of Community Medicine and Rehabilitation, Umeå University, Umeå, Sweden; Department of Psychology, Umeå University, Umeå, Sweden; Department of Community Medicine and Rehabilitation, Umeå University, Umeå, Sweden; Department of Psychology, Umeå University, Umeå, Sweden

**Keywords:** Assessment, Attention, Learning and Memory, Executive functions

## Abstract

**Objective:**

Neuropsychological testing has traditionally been performed on site using standardized paper-pencil tests. Online platforms now offer the potential of conducting such testing at home but requires validation before widespread use. In this pilot study with healthy adults, we examine the convergent validity of the newly developed test battery Mindmore Remote.

**Method:**

Fifty-two healthy participants were tested using both Mindmore Remote at home and traditional neuropsychological testing on site. The order of presentation was randomized. Associations between test performance on the two batteries were compared using Pearson and Spearman correlations.

**Results:**

Results revealed significant correlations between all Mindmore Remote tests and traditional tests. Verbal tests showed stronger correlations (*r* = .71–.83) than non-verbal tests (*r* = .48–.71). Further, correlations were stronger for users who made responses using a computer mouse than for touchpad users.

**Conclusions:**

Mindmore Remote tests that rely on verbal in- and output were comparable to traditional face-to-face neuropsychological tests. However, although promising, further validation is needed for tests that require visuo-motor interaction. In comparison with similar studies, the results indicate that test modification, rather than remote administration, is accountable for weaker correlations.

## INTRODUCTION

Neuropsychological assessment traditionally requires the patient to travel to a clinic, which can be challenging for patients with limited mobility, cognitive impairments, or those living in rural areas far from healthcare facilities. Conducting neuropsychological testing from home could eliminate the need to travel, and thereby increase access to neuropsychological assessment.

Both clinical and non-clinical populations seem satisfied with remote procedures in neuropsychological assessment (Abdolahi et al., 2016; Appleman et al., 2021; Gnassounou et al., 2022; Lacritz et al., 2020; Mahon et al., 2022). Patients report that doing the tests at home is both convenient and time efficient (Abdolahi et al., 2016; Barraclough et al., 2023; Lacritz et al., 2020). Similar sentiments are reported from healthcare staff, where remote assessment is regarded as a time and cost-saving alternative to traditional face-to-face testing (Luxton et al., 2014). In addition, remote digital testing counters some of the major weaknesses of traditional paper-pencil tests, such as administrator effects and scoring errors, especially regarding timing of tasks (Van Patten, 2021).

Despite the potential benefits, the clinical implementation of remote neuropsychological testing is still in its infancy. Concerns have been raised regarding the psychometric properties of remote tests, since deviations from the standardization and setting in which a test was developed might affect the psychometric qualities of the instrument (Barraclough et al., 2023; Bilder et al., 2020; Claessen et al., 2015). Previous research has stressed the need to evaluate more comprehensive remote test-batteries, calling for tests with standardized procedures of administration and scoring, but also with appropriate normative data for the remote assessment (Barraclough et al., 2023; Bilder et al., 2020; Brearly et al., 2017; Fox-Fuller et al., 2022; Gnassounou et al., 2022; Marra et al., 2020; Robinson & Brewer, 2016).

Mindmore (www.mindmore.se) is a digital application that meets several of these requirements. It comprises a test battery that aims to capture a broad range of cognitive functions. It is adapted for self-administration that can be done without the presence or assistance of a test administrator, with identical conditions for both instructions and stimuli presentation across test occasions. Furthermore, it provides automatic data storing and scoring. Mindmore tests can be performed at a clinic with a tablet or computer, or remotely with a computer. In the computerized version, both mouse and touchpad can be used as input device. Normative data has been collected for the tablet version (Van den Hurk et al., 2022) and the remote version (Mindmore, 2024b). Bjorngrim et al. (2019) validated a sub-sample of the tests in the tablet version, and did so on site. In this study, 81 healthy adults performed Rey Auditory Verbal Learning Test (RAVLT), Corsi Block, Paced Auditory Serial Addition Test, Trail Making Test (TMT), Stroop test, and Boston Naming test within the Mindmore platform on a tablet at one visit, and the correspondent traditional paper-pencil tests at the other. Significant correlations were found for each test measure, ranging from *r* = .34 to *r* = .67. The remote version of Mindmore have not been validated in the same manner, but a recent reliability study indicated satisfactory test–retest reliability for most measures within the Mindmore test battery in healthy adults (Bergman et al., 2025).

In summary, comprehensive remote test batteries could contribute to more equal opportunities in healthcare, for example by reducing administrator biases and by providing greater and faster access to neuropsychological assessment, especially for patients living in rural areas. Mindmore Remote is such a test battery, but needs to be validated before clinical implementation. Hence, our aim was to investigate the convergent validity of Mindmore Remote, comparing it to traditional, paper-based neuropsychological tests.

## METHODS

### Participants

Healthy volunteers were recruited through advertisement at the Umeå University campus and Umeå University Hospital, and through digital advertisements online. Those who expressed interest in participation were contacted by telephone for an interview during which they answered questions regarding inclusion and exclusion criteria. The inclusion criteria were (1) being 18 years of age or older and (2) having access to a computer with microphone, audio, and internet connection. Exclusion criteria were (1) any neurologic or neurodegenerative disorder; (2) neurodevelopmental diagnosis; (3) alcohol or substance abuse; (4) severe psychiatric disorder, including psychotic disorders, bipolar disorder, severe depression and suicidality, or having been treated as an inpatient at a psychiatric clinic during the past 12 months; (5) non-fluent level of Swedish or having lived in Sweden for less than 10 years; (7) medication that could affect cognitive performance and (8) being unable to undergo neuropsychological examination or provide informed consent due to physical or intellectual disability. All criteria were based on self-report. The study was approved by the Swedish Ethical Review Authority (issue number: 2022–06230-01) and performed in adherence with the World Medical Association’s Declaration of Helsinki and the study protocol (ClinicalTrials.gov NCT05819008; Berginstrom & Andersson, 2024). Sample size was determined on the basis of the power calculation in the protocol.

### Procedure

The study was a cross-sectional study with a randomized cross-over design. Participants were randomly assigned to perform the digital remote neuropsychological test-battery, Mindmore Remote, followed by a traditional paper-based neuropsychological testing—or vice versa. Recruitment and data collection took place from September 2023 to January 2024. Participants received both oral and written information about the study and signed informed consent prior to testing. As compensation, they received a gift card worth 100 SEK and were also offered a verbal summary of their test results from the traditional testing.

Mindmore Remote was self-administered and performed at home using the participants’ own computers. Participants received an email with a link to the test. After following the link, they were prompted to state whether they used a touchpad or mouse as input device. A test of the computer’s audio and audio recording was performed, and participants were asked to confirm that they heard the instructions properly. Before continuing with the tests, they were informed that the testing would take ~30 min with no opportunities for a break. They were instructed to take the test alone in an environment free from distractions, without the use of external aids, and to turn off their phones. The tests were then presented in a specific order, presented below.

The traditional testing was conducted at the University Hospital in Umeå, in a quiet room free from distractions, suitable for neuropsychological testing. The tests were administered by psychologists and trained psychology students in accordance with the standardized procedures described in the test manuals. The traditional tests were presented in the same order as their corresponding tests in Mindmore Remote. Participants filled out self-report questionnaires after the testing was completed. The two test sessions took place within two weeks, at least one day apart and approximately at the same time during the day for both sessions.

### Instruments

#### Self-assessment scales

Participants completed the self-report questionnaires Hospital Anxiety and Depression Scale (HADS) and Insomnia Severity Index (ISI). HADS consists of two subscales that aim to identify symptoms of depression and anxiety (Zigmond & Snaith, 1983). ISI intends to measure perceived insomnia in the last two weeks (Morin, 1993; Morin et al., 2011). Preceding the neuropsychological tests in Mindmore Remote, participants rated (1) how well they saw the text on the screen, (2) how well they heard, (3) how rested they felt and (4) how calm and free from stress they were. The self-assessment scale went from zero (Not good at all/Not at all) to five (Very/Very good).

#### Neuropsychological measures

**Fig. 1:**
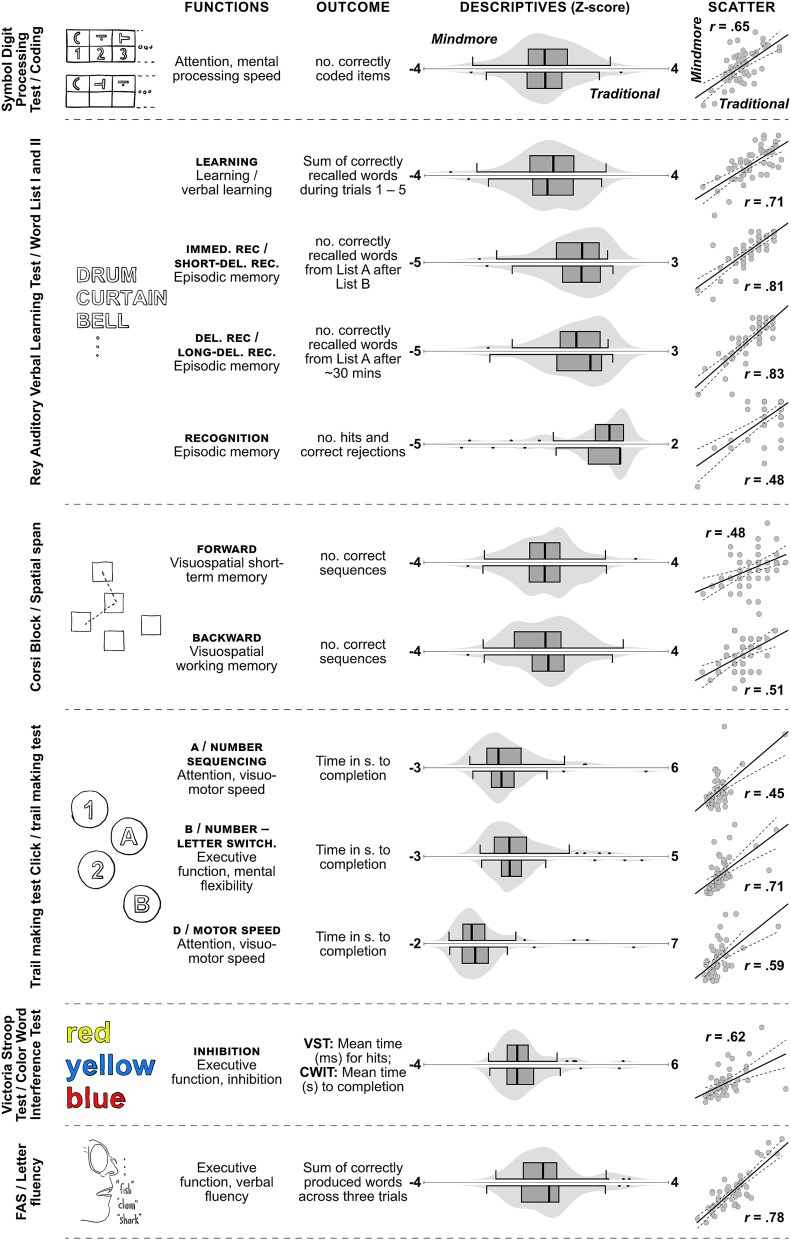
Main outcomes and visualization of results.

The tests in Mindmore Remote are described below, in the same order as they were administered in the testing session. Outcome measures and main cognitive functions captured by each test are presented in Fig. 1.

Symbol Digit Processing Test (SDPT): A digit-symbol code key is presented at the upper part of the screen. It contains nine different symbols and the numbers 1–9. Each symbol is associated with one of the nine digits. At the lower part of the screen is a 3 × 3 matrix with the numbers 1–9. One symbol at a time will appear at the center of the screen and the task is to, by using the code key, click at the digit in the matrix that is associated with that symbol. The test runs for 90 s, and participants are instructed to work as quickly and correct as possible. SDPT is validated against Coding from Wechsler Adult Intelligence Scale – Fourth Edition (WAIS-IV; Wechsler, 2008). Reliability coefficients of *r* = .86 was found in the normative study for Coding (Wechsler, 2008).

RAVLT: The test contains four parts: Learning, Immediate Recall, Delayed Recall and Recognition. The participant will hear a list of 15 words (List A). The task is to verbally recall as many words as possible immediately after hearing the list (Learning). The procedure is repeated for five trials, followed by a distraction list with 15 new words (List B). The participant is then asked, without hearing the words again, to recall the words from List A (Immediate Recall). This instruction is repeated after 30 min (Delayed Recall), followed by the task of identifying words from List A in a list of 30 words (Recognition). Delayed Recall and Recognition were performed after the Victoria Stroop Test described below. RAVLT is validated against Word List Recall I and II from Wechsler Memory Scale III (WMS-III; Wechsler, 1997b). The following test–retest coefficients were displayed in the normative study of WMS-III: .61–.72 for Recall Total Score, .62–.67 for Delayed Recall and .63–.66 for Recognition (Wechsler, 1997a).

Corsi Block Tapping Test (CBTT): The test consists of CBTT Forward and CBTT Backwards. In both, nine squares are presented at random locations on the screen. A few of them flash in a certain sequence, starting with two squares. In CBTT Forward, the participant is instructed to reproduce the sequence by clicking the squares in the same order as they flashed. In CBTT Backwards, the sequence should be reproduced in the reverse order. The test is validated against Spatial Span from WMS-III (Wechsler, 1997b) with a test–retest coefficients of .59–.62 in both the forward and the backward condition (Wechsler, 1997a).

Trail Making Test Click (TMT Click): The test consists of three subtests: TMT-A, TMT-B and TMT-D. Each subtest consists of 25 circles that must be clicked in the correct order, as quickly as possible. In TMT A, each circle contains a number from 1 to 25 and must be clicked in ascending order. In TMT B, each circle contains a number (1–13) or a letter (A-M). These must be clicked in ascending numerical and alphabetical order, but alternately (1-A-2-B-3-C). The circles in TMT D are blank and connected by a line that shows the way in which the circles must be clicked. The subtests are validated against their correspondent subtest from the Trail Making Test (TMT) in Delis-Kaplan Executive Function System (D-KEFS; Delis et al., 2001). In the normative study of TMT, the following test–retest correlations were found: .59 for Number Sequencing, .38 for Number-Letter Switching and .77 for Motor Speed (Delis et al., 2001).

Victoria Stroop Test (VST): Four buttons are presented at the lower part of the screen. The buttons are labelled “green”, “red”, “blue”, and “yellow”. A color word (green, red, blue, or yellow) is then presented at the center of the screen. The task is to click the button below that corresponds to the word that appeared. In Condition I, the font color is the same as the color word. In Condition II (Inhibition), the font color differs from the color word, and the instructions to click the button that matches the font color of the word. A total of 20 words are presented in each condition and the task should be done as quickly and correctly as possible. VST Inhibition is validated against the Inhibition subtest from the Color-Word Interference Test (CWIT) in D-KEFS (Delis et al., 2001). CWIT Inhibition had a test–retest correlation of .75 in its normative study (Delis et al., 2001).

FAS Word Fluency Test (FAS): The participant is presented with a letter followed by 60 s to verbally produce as many words as possible that begins with that letter. Repeated words, names and numbers are not allowed. The task is repeated three times, with a new letter for each trial (F, A, and S). FAS is validated against the subtest Letter Fluency from the Verbal Fluency Test in D-KEFS (Delis et al., 2001). A reliability coefficient of .80 was displayed in the normative study for Verbal Fluency (Delis et al., 2001).

### Statistical analyses

Statistical analyses were performed using PASW Statistics version 18.0. Descriptive analyses were conducted to report sample characteristics for the total sample, the group that used touchpad as input device, and the group that used mouse as input device. Independent sample *t*-tests were performed to assess statistical differences in age and years of education between the two groups (touchpad/mouse), and a chi-square test to investigate statistical differences regarding sex. One-sample *t*-tests were performed to determine if participants differed from the normative groups within Mindmore Remote and the traditional tests. The relationships between the tests in Mindmore Remote and their correspondent traditional tests were investigated using Pearson’s correlation coefficients for normal distributed data, and Spearman’s rank correlation coefficients when data was not normally distributed. This was decided using visual inspections of histograms. For the tests that required visuo-motor-responses (SDPT, CBTT Forward and Backwards, TMT Click and VST Inhibition), Pearson’s correlation or Spearman’s rank correlation were conducted for the group that used touchpad respectively mouse as input device. Raw scores were used for all correlation analyses. Results were considered statistically significant with a *p*-value less than .05.

## RESULTS

### Participants

Seventy individuals expressed interest in participating. Eleven were excluded due to fulfilling one or more exclusion criteria, resulting in 59 eligible participants. Six of them dropped out after scheduling, either before (*n* = 3) or after (*n* = 3) their first testing. After the second test appointment, one participant was excluded due to fulfilling an exclusion criterion. No participant was unable to perform the Mindmore test on their own computer in their home. Demographic data and results from self-assessment scales for the final 52 participants included in the analyses are presented in Table 1. Participants’ mean ratings of their physical state prior to Mindmore Remote indicated that they had good hearing (*M* = 4.15, *SD* = 1.42), and vision (*M* = 4.77, *SD* = 0.51), felt rested (*M* = 4.00, *SD* = 0.82) and free from stress (*M* = 3.77, *SD* = 1.10) before the remote testing.

**Table 1:** Demographic Characteristics for the Total Sample

	*n* (%)	*M*	*SD*	Range
Age	52	46.1	17.7	70 (19–89)
Education in years	52	15.8	2.86	18 (8–26)
Employment				
Full-time employee	35 (67.3)			
Part-time employee	4 (7.7)			
Student	6 (11.5)			
Retired	7 (13.5)			
Sex				
Male	24 (46.2)			
Female	28 (53.8)			
Test presentation				
Mindmore/Traditional	24 (46.2)			
Traditional/Mindmore	28 (53.8)			
Input device				
Mouse	33 (63.5)			
Touchpad	19 (36.5)			
Days between test	52	5.27	3.58	15 (1–16)
Hours of sleep^1^	52	7.02	1.21	6 (4–10)
HADS				
Anxiety scale	52	5.04	3.04	13 (0–13)
Depression scale	52	2.81	2.42	11 (0–11)
ISI	51	6.14	4.46	19 (0–19)

*Note. M* = Mean; *SD* = Standard Deviation; HADS = Hospital Anxiety and Depression Scale; ISI = Insomnia Severity Index.

^1^Hours of sleep the night before the traditional testing.

One outlier was excluded from the correlation analysis of CBTT Backwards. One participant shut off the audio recording during Mindmore Remote, causing missing data from FAS. Additional data loss was caused by technical issues in Mindmore Remote (RAVLT, FAS and VST) for one participant, and incorrect administration by the test leader on TMT Number-Letter Sequencing for one participant and TMT Motor Speed for one participant. There were no significant differences in age or education between the groups that used a touchpad or mouse as input device. There was however a significant difference regarding sex and input device, where significantly more women (53%) than men (17%) used touchpad as input device, *X*^2^ (1, *N* = 52) = 7.59, *p* = .006.

### Neuropsychological test results

Results from the neuropsychological test, both raw scores and standardized scores (Index score for Mindmore Remote and Scaled Scores for the traditional tests) are presented in Table 2. One-sample *t*-test revealed that participants scored significantly higher than the normative groups on all traditional tests (all *p <* .001). For the Mindmore tests participants differed significantly from the normative groups on two tests: SDPT where participants scored lower than the normative group (*p* = .002); and FAS, where participants scored higher than the normative group (*p* = .007).

**Table 2:** Neuropsychological Test Data and Results from One-sample t-test in Comparison with Normative Groups

	Mindmore Remote						Traditional Tests					
	*n*	Raw Score *M*	Raw Score *SD*	Index Score *M*	Index Score *SD*	*p*	*n*	Raw Score *M*	Raw Score *SD*	Index Score *M*	Index Score *SD*	*p*
SDPT – Coding	52	43.46	9.74	94.29	13.34	.002	52	71.62	14.06	109.70	12.75	<.001
RAVLT – Word List I and II												
Learning – Learning	51	52.98	10.77	104.55	17.69	.036	52	35.54	6.40	110.30	17.65	<.001
Immediate Recall – Short-Delayed Recall^1^	50	11.18	3.58	102.30	17.39	.177	52	9.00	2.42			
Delayed Recall – Long-Delayed Recall	51	11.02	3.82	102.14	17.27	.190	52	8.77	2.74	116.05	11.50	<.001
Recognition – Recognition	51	13.22	2.48	102.29	12.76	.103	52	23.33	1.10	107.80	9.55	<.001
Corsi Block – Spatial Span												
Forward – Forward	52	9.10	2.02	99.67	14.23	.435	52	9.10	1.98	107.30	16.00	<.001
Backwards – Backwards	51	8.04	1.96	99.65	13.89	.428	52	8.87	1.91	114.50	14.40	<.001
TMT Click – TMT												
TMT A – Number Sequencing	52	34.31	13.29	99.35	16.55	.388	52	27.35	14.81	112.70	10.70	<.001
TMT B – Number-Letter Switching	52	47.60	19.35	101.87	15.48	.194	51	69.59	36.10	108.25	13.40	<.001
TMT D – Motor Speed	51	21.58	6.67	97.33	17.46	.140	51	24.94	13.21	109.20	8.65	<.001
VST Inhibition – CWIT Inhibition	51	1564.96	474.22	99.88	14.87	.478	52	48.12	10.73	111.55	11.05	<.001
FAS – Letter Fluency	50	50.92	15.57	106.04	16.75	.007	52	53.23	14.94	121.85	17.00	<.001

Correlation coefficients and *p*-values for all analyses regarding relationships between Mindmore Remote tests and their corresponding traditional tests are presented in Table 3, and visually presented in Fig. 1. For the total sample, significant relationships (*p ≤* .001) were found for all outcome measures. The correlations were also significant (all *p* > .005) for the seven outcome measures analyzed in the group that used mouse as input device. In the group that used a touchpad as input device, correlations were significant (*p* < .05) for all tests, except VST Inhibition (*p* = .167), see Table 3.

**Table 3:** Correlations between Mindmore Remote and Corresponding Traditional Neuropsychological Test for the Total Sample and Samples Using Touchpad or Mouse as Input Device

Tests Correlated	All (*N* = 52)			Touchpad (*N* = 19)			Mouse (*N* = 33)		
	*n*	*r*	*p*	*n*	*r*	*p*	*n*	*r*	*p*
SDPT – Coding	52	.653 (P)	< .001^*^	19	.480 (S)	.037^*^	33	.631 (S)	< .001^*^
RAVLT – Word List I and II									
Learning – Learning	51	.707 (P)	< .001^*^						
Immediate Recall – Short-Delayed Recall	50	.810 (S)	< .001^*^						
Delayed Recall – Long-Delayed Recall	51	.830 (S)	< .001^*^						
Recognition – Recognition	51	.480 (S)	< .001^*^						
Corsi Block – Spatial Span									
Forward – Forward	52	.477 (P)	< .001^*^	19	.517 (P)	.024^*^	33	.476 (P)	.005^*^
Backwards – Backwards	51	.510 (P)	< .001^*^	19	.498 (P)	.030^*^	32	.515 (P)	.003^*^
TMT Click – TMT									
TMT A – Number Sequencing	52	.453 (S)	.001^*^	19	.497 (S)	.030^*^	33	.511 (S)	.002^*^
TMT B – Number-Letter Switching	51	.705 (S)	< .001^*^	19	.545 (S)	.016^*^	32	.757 (S)	< .001^*^
TMT D – Motor Speed	51	.590 (S)	< .001^*^	18	.517 (S)	.028^*^	33	.638 (S)	< .001^*^
VST Inhibition – CWIT Inhibition	51	.621 (S)	< .001^*^	18	.340 (S)	.167	33	.694 (S)	< .001^*^
FAS – Letter Fluency	50	.778 (S)	< .001^*^						

*Note.* Correlations are calculated from raw scores; CWIT = Color Word Interference Test; RAVLT = Rey Auditory Verbal Learning Test; SDPT = Symbol Digit Processing Test; TMT = Trail Making Test; VST = Victoria Stroop Test. (P) = Pearsons’s correlation; (S) = Spearman’s correlation.

^*^= statistically significant result.

## DISCUSSION

The aim of this study was to examine the convergent validity of Mindmore Remote. The results showed significant correlations between all the analyzed tests in Mindmore and their traditional counterpart, although to a varying degree.

The correlation coefficient between the mental processing speed tests SDPT and Coding was *r* = .65, which is lower than the .86 test–retest coefficients for Coding. A weaker correlation could be expected since SDPT and Coding varies in both stimuli presentation and procedure. SDPT presents one stimulus at a time and is performed by clicking the correct digit, while Coding presents all stimuli at once and requires written responses. This could place different demands on the cognitive functions needed to solve the task, such as visual short-term memory, learning abilities, visuo-motor-speed, and coordination (Wechsler, 2008) – causing a weaker correlation than the test–retest for Coding. Previous research supports this hypothesis. For example, verbal versions of mental processing speed tests conducted remotely have displayed correlations of *r* =. 0.80–0.88 when compared to traditional versions (Barcellos et al., 2018; Eilam-Stock et al., 2021; Levy et al., 2023). This indicates that the test presentation, rather than the remote administration, explains the weaker correlation in the present study. Furthermore, Atkins et al. (2022) found stronger correlations (*r* = 0.72–0.74) when validating a digital coding task that unlike SDPT shared layout with the traditional version – thus eliminating some variables that might influence visual learning and memory processes. In conclusion, results from the current study are promising but since SDPT and Coding might involve somewhat different cognitive processes, this calls for further psychometric evaluations of SDPT.

The correlations between RAVLT and Word List I and II in the present study were stronger than those found in Bjorngrim et al. (2019) that compared performances on a tablet version and the traditional version of RAVLT. Results for RAVLT in the present study align with results found by Barcellos et al. (2018) that validated a remote, web-based version of another word list test, the California Verbal Learning Test—Second Edition (CVLT-II) (Delis et al., 1987-2000), a test that highly resembles RAVLT. A significant correlation of *r* = .71 for Immediate Recall was found in their study as well as the current study. Furthermore, in comparison to the test–retest coefficients for Word List I and II, the correlation coefficient in the current study were consistent with the test–retest coefficients for Immediate Recall (*r* = .71 versus *r* = .61–.72), higher for Delayed Recall (*r* = .83 versus *r* = .62–.67) and lower for Recognition (*r* = .48 versus *r* = .63–.66). The relatively weak correlation for Recognition appears in several studies (Delaney et al., 1992; Magalhaes et al., 2012; Stricker et al., 2021) and is most likely explained by a low dispersion in the outcome measure rather than low equivalence with correspondent traditional tests. Overall, results indicate that remote administration is at least as valid as on-site, tablet administration and appears comparable to a traditional face-to-face version.

The current study is, to our knowledge, the first to investigate the convergent validity of a self-administered, computerized version of CBTT conducted remotely. Correlations between CBTT and Spatial span were close to .50 for both conditions in the current study. This correlation is slightly lower than the test–retest correlations in the normative study of Spatial Span (.60–.62 forward, .59–.62 backwards), but resembles the results found by Bjorngrim et al. (2019). Robinson and Brewer (2016) and Siddi et al. (2020) also investigated correlations between Spatial Span and tablet version of CBTT. Robinson and Brewer (2016) found a correlation of *r* = .39 for span length in a forward condition. Siddi et al. (2020) found a correlation of *r* = .50 in the forward condition, and *r* = .70 backwards. This suggests that the computerized version of CBTT in Mindmore Remote does not deviate from on-site tablet versions of CBTT. However, when comparing CBTT to Spatial Span, careful consideration is crucial. While the two tests set out to measure the same underlying cognitive processes, it has been demonstrated that timing in stimuli presentation and movement-related information can activate different cognitive processes and thereby influence performances in CBTT (Brunetti et al., 2014). CBTT in Mindmore Remote lacks this timing and movement-related information, but is provided in Spatial Span. While the blocks flashes with an even inter-stimuli interval in CBTT, the test-leader in Spatial Span tap the blocks by moving the hand between the blocks. The latter (1) creates a movement-related trajectory and (2) makes the inter-stimuli interval congruent with the spatial distances between blocks, both of which seem to enhance performances (Brunetti et al., 2014). The fact that CBTT in Mindmore Remote differs from Spatial Span according to these aspects could explain the relatively low agreement between the two test versions. The tests might capture somewhat different cognitive underlying processes. This highlights the importance of psychometric evaluations when changing the mode of an established test.

The correlation between Click TMT-A and TMT Number Sequencing were slightly lower (*r* = .45) than the test–retest coefficients for TMT Number Sequencing (*r* = .59). This was also true for Click TMT-D and TMT Motor Speed (*r* = .59) when compared to test–retest correlation for TMT Motor Speed (*r* = .77). In contrast, the correlations between Click TMT-B and TMT Number-Letter Switching were stronger (*r* = .71) than the test–retest correlation for TMT Number-Letter Switching (*r* = .38), indicating that differences in motor-response (clicking versus drawing lines) cannot explain the weaker correlation for Click TMT A and D. Previous research has found varying results when comparing results on traditional TMT tests with tablet versions conducted on site. For TMT B, correlations have ranged from non-significant (Karimpoor et al., 2017) or relatively weak (Amirpour et al., 2024) to relatively strong (Fellows et al., 2017; Park & Schott, 2022). Furthermore, Vermeent et al. (2022) found that performance on TMT B was influenced by participants experience with digital devices, which was not the case for TMT A. In conclusion, the results for TMT in the current study are promising but challenging to interpret. Together with the inconsistent results found in previous studies, caution is warranted in concluding equivalence between TMT Click in Mindmore Remote and the traditional TMT.

The strength of the correlation between VST Inhibition and CWIT Inhibition (*r* = .62) were similar to the one found in Bjorngrim et al. (2019) (*r* = .66), but slightly weaker than the test–retest correlation for CWIT Inhibition in the normative study of D-KEFS (*r* = .75). This could be expected due to the differences between VST in Mindmore Remote and CWIT. In CWIT, all words are organized in rows and presented simultaneously while VST presents one stimulus at a time at the center of the screen. Salo et al. (2001) found that participants reaction times in Stroop Inhibition were facilitated by presenting one stimulus at a time compared to the traditional simultaneous presentation, hypothesizing that the single-stimulus presentation reduced the strain in shifting attention and thereby decreasing the reaction time. Another difference between the remote and traditional test is that verbal responses are required in CWIT and a non-verbal motor-response in VST. Changing a test from written to verbal and vice versa can influence performance (Barraclough et al., 2023). Further research is needed to better understand the cognitive processes that might be influenced by the modifications made from the traditional to the digital remote version, but results from Salo et al. (2001) indicate that a single-stimuli presentation (like the one in VST) might reduce the influence of attention-shifting processes in the task—possibly isolating the inhibition-ability more effectively than CWIT. When these differences are considered, the results from the current study suggest that the remote VST displays good agreement with the traditional CWIT.

The current study found a significant relationship of *r* = .78 for the verbal fluency test FAS, which is the same strength of correlation found by Fox-Fuller et al. (2022) in a study that administered FAS twice in a remote setting using videoconference technology. Results also resembles the test–retest coefficients of .80 for Letter Fluency in D-KEFS (Delis et al., 2001). Results adds to the previous research that has found strong support for the validity of letter fluency tests in tele-neuropsychology assessment (Atkins et al., 2017; Atkins et al., 2022; Marra et al., 2020). Thus, the remote version of FAS is shown to be a valid alternative to the face-to-face version.

We also investigated the correlation between the test versions with regard to form of input device (mouse respectively touchpad). In general, the strengths of the correlations were comparable, or in favor for the group that used a mouse. Correlations remained significant in all cases except for VST in the group that used a touchpad. This non-significant result and weaker correlations for the touchpad group could be caused by a loss in statistical power due to a relatively small sample size. However, it could also be that the use of a mouse facilitates performances. This was observed in the study that collected normative data for Mindmore Remote, where the use of a touchpad had a negative impact on time dependent tests, including VST (Mindmore, 2024a). Thus, it has been recommended to use a mouse when performing the tests in Mindmore Remote. Results from the current study align with that conclusion, since the weakest correlations for several test were found for participants using a touch pad.

In comparison with data from normative groups, participants in the present study scored significantly higher than expected based on the normative data from the traditional tests. However, when compared to normative data from Mindmore Remote, participants performed significantly different in only two tests. There are several possible explanations for the differences from normative groups in the traditional tests. The normative data for the traditional tests are over 15 to 25 years old (Delis et al., 2001; Wechsler, 1997b, 2008). In addition, the Mindmore Remote normative data adjusts not only for age, but also level of education and gender. Since the participants in the present study were quite highly educated, this highlights the importance of using normative data that is both updated and adjusted for factors beyond just age.

### Limitations

While this study contributes valuable insights regarding remote administration of a neuropsychological test-battery, it contains limitations. The sample is relatively small, and a larger sample would allow for more sophisticated and reliable analyses of factors influencing performance on the Mindmore Remote. Since participants performed the remote test-battery in their home without the supervision of a test leader, the study could not detect any environmental disturbances that could have affected test performance. Furthermore, the non-supervision meant that the study lacked visual identification of the test taker, meaning that a participant could have let another person perform the test. However, the strong correlation coefficients for the verbal tests indicate that the same person performed both the traditional testing and the remote testing at home. In addition, it is important to recognize that motivation is an important factor in neuropsychological testing (Brown & Zakzanis, 2023). Thus, the lack of a test aimed at measuring motivation in the current study is another study limitation. Examining motivational factors by including performance validity test, as well as identification and ensuring of an undisturbed test environment, for example by video recording or web camera surveillance, are all important to consider before the test can be administered clinically. Ensuring a proper and disturbance free environment is indeed a significant challenge when using remote testing. This study did not collect any data on familiarity with computers or other digital devices. While no participant was unable to perform Mindmore on their own computer, generalizability to populations not in ownership of a computer, or less familiar with computers, might be somewhat limited. Since the participants were all Swedish speaking, the results are not generalizable to populations with other language or cultural backgrounds. This is a major limitation in the field of neuropsychology, that needs to be addressed more frequently (Franzen et al., 2022), including when conducting remote testing. This issue is therefore suggested as being an important venue of research in the future. Lastly, since this study only included healthy participants, the results are not generalizable to clinical populations. The cognitive difficulties of patients that are in need of a neuropsychological evaluation, such as patients with dementia or acquired brain injuries, may actually be a factor making remote testing inappropriate or impossible to perform. Specific studies investigating the test in clinical populations must be performed before Mindmore remote can be implemented in clinical practice.

### Conclusion and future directions

In conclusion, the tests in Mindmore Remote that rely on verbal in- and output (RAVLT and FAS) are deemed equivalent with traditional face-to-face neuropsychological tests. While the results are promising for the remote tests that require visuo-motor interaction (SDPT, CBTT, TMT Click and VST), the somewhat weaker correlations to the traditional test warrants further investigation before the same conclusion as for the verbal tests can be drawn. Still, in comparison with test–retest coefficients on the traditional tests, the correlations between Mindmore Remote test and their traditional counterpart are quite satisfactory. In comparison with similar convergent validity studies (i.e., Atkins et al., 2022; Bercellos et al., 2018), the results indicate that test modifications, rather than remote administration, account for weaker correlations, as test modifications can impact the cognitive processes involved in the task. Several studies (Atkins et al., 2022; Barraclough et al., 2023; Eilam-Stock et al., 2021; Fox-Fuller et al., 2022), including systematic reviews (Brown & Zakzanis, 2023) indicate that remote administration of neuropsychological tests can be both valid and reliable. Still, more studies regarding psychometric evaluation of tests are needed before clinical implementation in order to make valid interpretations. It is even possible that a digital, remote test succeeds better than a corresponding traditional test in capturing the main cognitive function it sets out to measure, this by reducing the influence of additional cognitive processes necessary for the test. These questions should be addressed in further research alongside validation of remote tests in the clinical populations that are in need of neuropsychological assessment. In addition, test validation can be performed in other ways than merely convergent validity studies. Including other measures, such as self-assessment scales of cognitive/executive problems, or more ecologically relevant measures, such as school performance, could be used. Future studies should also perform item analyses within all Mindmore Remote tests. Such validation studies, in both healthy and clinical populations, are critical to perform before the test can be implemented clinically.
